# Illumination Normalization of Face Image Based on Illuminant Direction Estimation and Improved Retinex

**DOI:** 10.1371/journal.pone.0122200

**Published:** 2015-04-23

**Authors:** Jizheng Yi, Xia Mao, Lijiang Chen, Yuli Xue, Alberto Rovetta, Catalin-Daniel Caleanu

**Affiliations:** 1 School of Electronic and Information Engineering, Beihang University, Beijing, 100191, China; 2 Department of Mechanics, Polytechnic University of Milan, Milan, 20156, Italy; 3 Applied Electronics Department, University POLITEHNICA Timisoara, Timisoara, 300223, Romania; Leibniz Institute for Age Research, GERMANY

## Abstract

Illumination normalization of face image for face recognition and facial expression recognition is one of the most frequent and difficult problems in image processing. In order to obtain a face image with normal illumination, our method firstly divides the input face image into sixteen local regions and calculates the edge level percentage in each of them. Secondly, three local regions, which meet the requirements of lower complexity and larger average gray value, are selected to calculate the final illuminant direction according to the error function between the measured intensity and the calculated intensity, and the constraint function for an infinite light source model. After knowing the final illuminant direction of the input face image, the Retinex algorithm is improved from two aspects: (1) we optimize the surround function; (2) we intercept the values in both ends of histogram of face image, determine the range of gray levels, and stretch the range of gray levels into the dynamic range of display device. Finally, we achieve illumination normalization and get the final face image. Unlike previous illumination normalization approaches, the method proposed in this paper does not require any training step or any knowledge of 3D face and reflective surface model. The experimental results using extended Yale face database B and CMU-PIE show that our method achieves better normalization effect comparing with the existing techniques.

## Introduction

Many objective factors restrict the development of face recognition (FR) and facial expression recognition (FER) systems, such as face posture, illumination variation, and so on. Some works [1, 2] have pointed out that the changes caused by the variation of illumination could be more significant than the differences between individual’s physical appearance. Furthermore, some researchers [3] affirm that the variation of illumination could bring more negative influence to FR comparing with the pose and expression. Removing the negative effects of illumination over FR and FER is a challenging problem in image processing which generates intensive research efforts. The relevant work in the field [4–9] could be briefly divided into three categories: face modeling, invariant features extraction, and illumination normalization. Face modeling assumes some face images with same pose but with different illumination condition. In this way, a low-dimensional illuminant subspace is created and the main idea is to find out the degree of illumination variation in the low-dimensional illuminant subspace. In order to diminish or even eliminate the negative effects of illumination variations over FR and FER, the method of invariant features extraction focuses on extracting those features which are insensitive to illumination variation from face images. The method of illumination normalization preprocesses face images and aims to obtain face images with uniform illumination. This method does not require any training images, prior knowledge of the 3D face models or the reflection parameters. Moreover, its calculation process is relatively simple. These advantages make illumination normalization the preferred solution for removing the negative effects of illumination over FR and FER. Some relevant works are briefly summarized below.

Histogram Equalization (HE) [10] usually increases the global contrast of many face images and often produces realistic effects in those face images with backgrounds and foregrounds that are both bright or both dark. However, this method fails in images with backgrounds and foregrounds that are quite different, such as photographs. There are also a number of improved HE algorithms, such as Block-based Histogram Equalization (BHE) [11], Adaptive Histogram Equalization (AHE) [12], Oriented Local Histogram Equalization (OLHE) [13], and so on. Local Normalization Technology (LNT) [14] can effectively eliminate the negative effect of uneven illumination while keeping the local statistical properties of the processed image the same as in the corresponding image under normal illumination condition. Xie and Lam [15] use both LNT and HE to eliminate the effects of nonuniform illumination, resulting higher accuracy face recognition rates. Ruiz-del-Solar and Quinteros [16] apply the Illumination Plane Subtraction (IPS) together with HE and in order to reduce shadows caused by extreme lighting angles. In their paper, they have given some experimental results and claimed that IPS together with HE could be applied in face detection and recognition. Before applying the illumination compensation and normalization algorithms, they apply other pre-processing stages to obtain aligned face images of uniform size. IPS together with HE is evaluated in a face identification scenario (1 against n comparison). In the first stage of their study, IPS together with HE was applied in 16 different face recognition systems, which are built using four different projections methods and four different similarity measures. The recognition rates can be found in table 1 of their paper [16]. Emadi et al. [2] conclude that the illumination components reside in the low frequency sub-band. In their method, an input face image is decomposed into its high frequency and low frequency components. After setting low frequency components to zero and approximating new coefficients, face image is reconstructed by wavelet inverse transform. Self-Quotient Image (SQI) [17, 18] is based on the Quotient Image (QI) [19] method and has become an important illuminant normalization method for FR and FER. Wang et al. [20] introduce a nine-dimension face illumination subspace based on QI and construct a lower-dimension training matrix. Thus, they synthesize the nine typical illuminant samples and standard illuminant samples, and implement the illumination normalization of the gray and the color images. Based on the mathematical foundations of Land’s Retinex theory, Jobson et al. define a practical implementation of the Retinex without particular concern for its validity as a model for face imagery and color perception and present the Single-Scale Retinex (SSR) algorithm [21] and the Multi-Scale Retinex (MSR) algorithm [22]. In order to achieve illumination invariant eye detection under varying lighting conditions, Jung et al. [23] use adaptive smoothing based on the Retinex theory to remove the illumination effects. To some extent, although the MSR algorithm can partially reduce the ‘halo’ phenomenon of SSR algorithm, the result of illumination normalization is still not ideal. Local Binary Pattern (LBP) [24] is a typical illumination invariant feature extraction method. Cheng et al. [25] propose Local Binary Patterns Image (LBPI), which uses the LBP texture descriptors to every point of face image and combines the LBP features for all points into an image, to improve the performance of face recognition under various illumination conditions. Bayu and Miura [26] create an adaptive contrast ratio based on Fuzzy by considering two models (appearance estimation model and shadow coefficient model) of individual face as input, and then apply a Genetic Algorithm to optimize the Fuzzy’s rule. They use Principal Component Analysis (PCA) and Nearest Neighbor (NN) based on correlation distance as the classifiers, and the experimental result shows that their algorithm is robust enough in order to normalize uneven illumination moderate and hard illumination [26]. For avoiding the ‘halo’ phenomenon, Yang et al. [27] proposes a face recognition method under the condition of illumination changes based on the improved the Retinex algorithm. The experimental results show that the improved Retinex algorithm reduces the time complexity and gets good image enhancement effect, and it can be a better solution to the ‘halo’ issue [27]. In addition, there are some algorithms [28–30] claim that they can provide good illumination normalization effect.

Although all the methods mentioned above can diminish or eliminate the impact of uneven illumination in face images, still some drawbacks persist: the need for training images, prior knowledge of the face models, reflection parameters, etc. It is known that the face images will appear different under different illumination directions: face regions which are closer to the light source tend to be brighter and the ones which are farther from the light source have the opposite characteristic. Our proposed method estimates the illuminant directions of face images and takes advantage of the improved Retinex algorithm. The experiments are performed using the extended Yale face database B and CMU-PIE. The results show that the proposed method can restore the original texture information and structure characteristic, and achieve more favorable normalization effect comparing with the existing techniques.

The rest of the paper is organized as follows. In Section 2, the framework and details of our proposed illuminant normalization method are given. In Section 3, the experimental results and analysis are performed on the databases whereby the proposed method is evaluated and compared to other methods. Finally, conclusions and discussions are presented in Section 4.

## The Proposed Method

**Fig 1:**
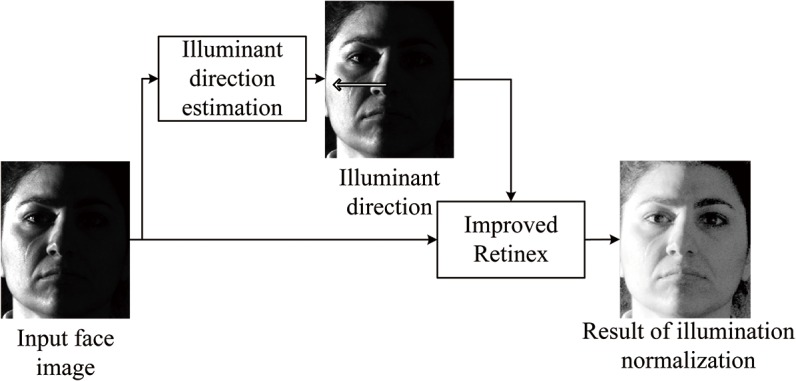
System architecture for the proposed illuminant normalization method. Reprinted from http://vision.ucsd.edu/~leekc/ExtYaleDatabase/ExtYaleB.html under a CC BY license, with permission from David Kriegman, original copyright 2001.

The system architecture of the proposed illuminant normalization method is shown in Fig 1, which includes two parts: illuminant direction estimation of the input face image and illumination normalization based on improved Retinex. The basic procedure of the proposed method is that we firstly estimate the illumination directions of face images based on local region complexity analysis and average gray value, then we improve the Retinex algorithm considering the results of illuminant direction estimation, and finally we cope with the problem of illumination normalization.

### 2.1 Illuminant Direction Estimation

The illuminant direction estimation method we applied in this paper was formulated in our previous work [31], and the reasons why we need to estimate the illuminant directions of face images for illumination normalization are given in Subsection 2.2.

**Fig 2:**
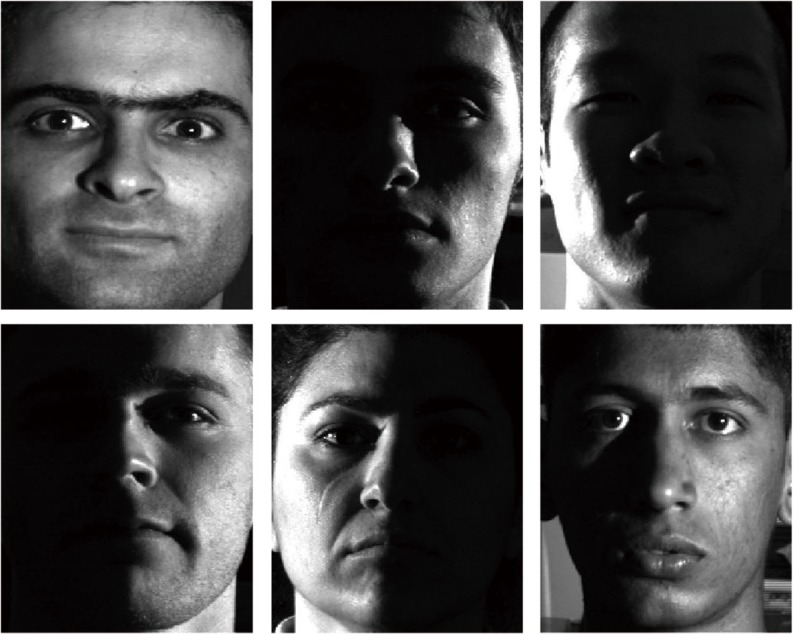
Some examples of different illumination distributions. Reprinted from http://vision.ucsd.edu/~leekc/ExtYaleDatabase/ExtYaleB.html under a CC BY license, with permission from David Kriegman, original copyright 2001.

**Fig 3:**
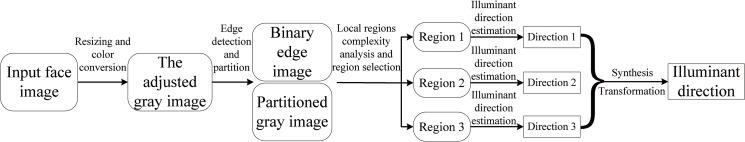
The main steps of the illuminant direction estimation method. It should be noted that three of sixteen local regions are selected to calculate the final illuminant direction.

Different regions in face images make different contributions to illuminant direction estimation. For example, the smooth regions play a more important role than the concavo-convex ones [32]. Usually, as illustrated in Fig 2, the smooth regions have similar textures and simple edges, whereas the concavo–convex regions have opposite characteristics. So it is worthwhile to find out regions with simple edges for illuminant direction estimation. The system architecture of the illuminant direction estimation method is shown in Fig 3. In order to better describe our method, we list the basic six steps of the method, as shown below.

1. Adjust the sizes of face images (color or gray) to a uniform value. Color face images are transformed to YCbCr color space, and the luminance component Y is selected as the input gray image. Gray face images are directly utilized as the input.
2. Use the Canny edge detector to find object boundaries in the luminance component Y and get the binary edge image.
3. Divide the binary edge image and the luminance component Y into sixteen local regions. The reason why we split the image into exactly sixteen regions has been described in our previously published manuscript [31].
4. For each local region, analyze its complexity depending on the edge level percentage and calculate its average gray value.
5. Estimate the illuminant directions of the three selected local regions with less complexity and large average gray value. The reason why we choose exactly three regions has been described in our previously published manuscript [31].
6. Synthesize the three illuminant directions and transform the result to the final illuminant direction in the original face image (color or gray).

In all the steps mentioned above, the local region complexity analysis and illuminant direction estimation are particularly important. Chacon et al. [33, 34] present a description of image complexity analysis based on the edge level percentage which is defined by

ψ=|A|/(width×height)  A={p(x,y)|p(x,y)=1} 1

Where |•| indicates cardinality of a set, *p*(*x*,*y*) denote the gray value at pixel (*x*,*y*), and width × height is the dimension of the image. After receiving the edge level percentage of each local region, we calculate the average gray value of each local region, and then select three local regions with less complexity and large gray value for estimating the illuminant direction.

Model-based illuminant direction estimation is an effective and popular way to estimate a complex scene illumination. These models include Lambertian, dichromatic, Torrance-Sparrow models, and so on. Our proposed illuminant direction estimation method is inspired by model-based illuminant direction estimation and aims to achieve a higher recognition rate in less execution time.

The normal direction of a pixel could be seen as the direction in which the most dramatic change in gray values occurs, which means the points on the normal lines have maximal gray-scale difference from the center pixel. Sometimes, a plurality of points meets the requirements of the calculation process. For this case, the final normal direction is defined as the summation of normal lines, and the magnitude of the normal is the value of maximal gray scale, which is the difference between the points on the normal lines and the center pixel. So the normal direction of any pixel in the image could be calculated.

The *m* ^*th*^ and *n* ^*th*^ selected regions are represented as *f* ^*m*^(*x*,*y*) and *f* ^*n*^(*x*,*y*) (*m*,*n* = 1,2,3 and *m* ≠ *n*), respectively. Thus, the error function between the calculated intensity *M*_*v*_ and the measured intensity *b* is given by

$$\begin{array}{r} f_{1}\left(L^{m},L^{n},k_{\alpha }\right)=\|[\begin{array}{cccc} N_{x}\left(f^{m}\left(x_{1},y_{1}\right)\right) & N_{y}\left(f^{m}\left(x_{1},y_{1}\right)\right) & 0 & \\ N_{x}\left(f^{m}\left(x_{1},y_{2}\right)\right) & N_{y}\left(f^{m}\left(x_{1},y_{2}\right)\right) & 0 & \\ \vdots & \vdots & \vdots & \\ N_{x}\left(f^{m}\left(x_{p},y_{p}\right)\right) & N_{y}\left(f^{m}\left(x_{p},y_{p}\right)\right) & 0 & \\ 0 & 0 & N_{x}\left(f^{n}\left(x_{1},y_{1}\right)\right) & \\ 0 & 0 & N_{x}\left(f^{n}\left(x_{1},y_{2}\right)\right) & \\ \vdots & \vdots & \vdots & \\ 0 & 0 & N_{x}\left(f^{n}\left(x_{p},y_{p}\right)\right) & \ldots \end{array}\\ {\begin{array}{cc} 0 & 1\\ 0 & 1\\ \vdots & \vdots \\ 0 & 1\\ N_{y}\left(f^{n}\left(x_{1},y_{1}\right)\right) & 1\\ N_{y}\left(f^{n}\left(x_{1},y_{2}\right)\right) & 1\\ \vdots & \vdots \\ N_{y}\left(f^{n}\left(x_{p},y_{p}\right)\right) & 1 \end{array}]\left[\begin{array}{c} L_{x}^{m}\\ L_{y}^{m}\\ L_{x}^{n}\\ L_{y}^{n}\\ k_{\alpha } \end{array}\right]-\left[\begin{array}{c} f^{m}\left(x_{1},y_{1}\right)\\ f^{m}\left(x_{1},y_{2}\right)\\ \vdots \\ f^{m}\left(x_{p},y_{p}\right)\\ f^{n}\left(x_{1},y_{1}\right)\\ f^{n}\left(x_{1},y_{2}\right)\\ \vdots \\ f^{n}\left(x_{p},y_{p}\right) \end{array}\right]\|}^{2}={\left\|Mv-b\right\|}^{2} \end{array}$$ 2

Where ∥•∥ indicates the magnitude of a matrix, $L_{x}^{m}$and $L_{y}^{m}$ are two components of the illumination direction corresponding to the *m*th selected region, $L_{x}^{n}$and $L_{y}^{n}$ are two components of the illumination direction corresponding to the *n*th selected region, *N*_*x*_(*f* ^*m*^(*x*_*i*_,*y*_*i*_)) and *N*_*y*_(*f* ^*m*^(*x*_*i*_,*y*_*i*_)) are two components of the normal vector in pixel (*x*_*i*_,*y*_*i*_) of the *m*th selected region, *N*_*x*_(*f* ^*n*^(*x*_*i*_,*y*_*i*_)) and *N*_*y*_(*f* ^*n*^(*x*_*i*_,*y*_*i*_)) are two components of the normal vector in pixel (*x*_*i*_,*y*_*i*_) of the *n*th selected region, and *k*_*α*_ is the constant intensity of environmental light. Because the human face occupies a very small percentage of the entire illuminant scene, the face images are considered to meet the infinite light source model. Therefore, the constraint function is given by

$$f_{2}\left(L^{m},L^{n},k_{\alpha }\right)={\left\|\left[\begin{array}{ccccc} -1 & 0 & 1 & 0 & 0\\ 0 & -1 & 0 & 1 & 0 \end{array}\right]\left[\begin{array}{c} L_{x}^{m}\\ L_{y}^{m}\\ L_{x}^{n}\\ L_{y}^{n}\\ k_{\alpha } \end{array}\right]\right\|}^{2}={\left\|Cv\right\|}^{2}$$ 3

Thus, the final error function is defined as

$$f\left(L^{m},L^{n},k_{\alpha }\right)=f_{1}+\lambda f_{2}$$ 4

where λ is the Lagrange multiplier. So the process of calculating *v* is translated into seeking the optimal solution for the following system of equations:

$$\{\begin{array}{c} \min\;f_{1}\left(L^{m},L^{n},k_{\alpha }\right)={\left\|Mv-b\right\|}^{2}\\ s.t.\;f_{2}\left(L^{m},L^{n},k_{\alpha }\right)={\left\|Cv\right\|}^{2}=0 \end{array}$$ 5

Seeking the optimal solution for Eq. (5) is equivalent to solving the following equations:

$$\left\{ \begin{array}{l} \partial f/\partial v=2M^{\text{T}}Mv-C^{\text{T}}\lambda -2M^{\text{T}}b=0\\ Cv=0 \end{array}\right..$$ 6

For the constraint function being equal to zero, we know that *L* ^*m*^ is equal to *L* ^*n*^. Our approach assumes that *L*(*m*,*n*) represent *L* ^*m*^ or *L* ^*n*^. Supposing that (*m*,*n*) exists three particular cases ((1,2), (2,3), and (3,1)), the three illuminant directions (*L*(1,2), *L*(1,2), and *L*(1,2)) in a face image could be calculated by using Eq. (6).

Although *L*(1,2), *L*(1,2), and *L*(1,2) are often different from the actual illuminant direction, they are all useful and have different contributions to estimate the final illuminant direction. The edge level percentages can be used to adjust *L* by calculating

$$W\left(m,n\right)=\frac{1}{\psi_{m}+\psi_{n}}L\left(m,n\right).$$ 7

Let $L^{'}=\left(L_{x}^{'},L_{y}^{'}\right)$ represent the synthesized illuminant direction:

$$L^{'}=W(1,2)L(1,2)+W(2,3)L(2,3)+W(3,1)L(3,1).$$ 8

We assume the size of resized face image is *Q*×*Q*. The final transformed illuminant direction *L*_*final*_ is calculated by

$$L_{final}=\left(L_{final-x},L_{final-y}\right)=\left(L_{x}^{'}\cdot \text{width}/Q,L_{y}^{'}\cdot \text{height}/Q\right)$$ 9

**Fig 4:**
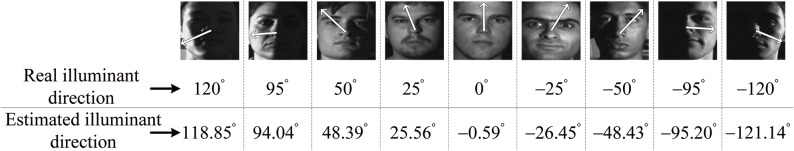
Real illuminant directions and estimated illuminant directions. Reprinted from http://vision.ucsd.edu/~leekc/ExtYaleDatabase/ExtYaleB.html under a CC BY license, with permission from David Kriegman, original copyright 2001.

Real illuminant directions and estimated illuminant directions are shown in Fig 4.

### 2.2 The Improved Retinex Theory

Retinex theory was described in 1971 by Land and McCann [35]. The word ‘Retinex’ is a portmanteau formed from ‘retina’ and ‘cortex’, suggesting that both the eye and the brain are involved in the processing. Retinex theory supposes that the image *S*(*x*,*y*) is the product of two different images, the reflectance *R*(*x*,*y*) with high frequency components and the illumination *L*(*x*,*y*) with low frequency components. The relationship between the three can be expressed as

S(x,y)=R(x,y)⋅L(x,y) 10

By estimating the illuminant component, illumination normalization based on Retinex theory decomposes the reflection component, thus eliminates the influence of uneven illumination to improve the visual effect of face image. Eq. (10) is usually transformed into logarithmic form

$$\begin{array}{l} \log[S\left(x,y\right)]=\log[R\left(x,y\right)\cdot L\left(x,y\right)]\;\\ \;=\log[R\left(x,y\right)]+\log[L\left(x,y\right)] \end{array}$$ 11

Let *s*(*x*,*y*), *r*(*x*,*y*), and *l*(*x*,*y*) represent log[*S*(*x*,*y*)], log[*R*(*x*,*y*)], and log[*L*(*x*,*y*)], respectively.

s(x,y)=r(x,y)+l(x,y) 12

Thus,

r(x,y)=s(x,y)−l(x,y) 13

That is to say, since *s*(*x*,*y*) is known, we can calculate *r*(*x*,*y*) by estimating *l*(*x*,*y*). Therefore, a reasonable and effective method for estimating *l*(*x*,*y*) is the key to illumination normalization based on Retinex theory.

Compared to the previous Retinex, the center/surround Retinex can significantly increase the operation speed and present better processing effect. SSR [21] is a typical center/surround Retinex method whose mathematical representation derives from Eq. (13).

r(x,y)=s(x,y)−l(x,y)=s(x,y)−log[S(x,y)*F(x,y)] 14

where ‘*’ denotes the convolution operation, and *F*(*x*,*y*) is the surround function. The surround function determines the type of SSR and has several forms:

$$F\left(x,y\right)=1/\left(x^{2}+y^{2}\right)$$ 15

$$F\left(x,y\right)=1/\left[1+\left(x^{2}+y^{2}\right)/c_{}^{2}\right]$$ 16

$$F\left(x,y\right)={\text{e}}^{-{\left(x^{2}+y^{2}\right)}^{1/2}/c}$$ 17

$$F\left(x,y\right)={\text{Ke}}^{-\left(x^{2}+y^{2}\right)/c_{}^{2}}$$ 18

where *c* is the scale parameter of surround function, and K is selected such that

∬F(x,y)dxdy=1. 19

For illumination normalization of face image, Eq. (19) has the discrete representation:

$$\sum_{x}\sum_{y}F\left(x,y\right)=1.$$ 20

Jobson et al. [21] have proved that SSR can present the best illumination normalization effect when the surround function is a Gaussian function. Let *G*(*x*,*y*) represent *F*(*x*,*y*), then we can rewrite Eq. (14):

r(x,y)=s(x,y)−l(x,y)=s(x,y)−log[S(x,y)*G(x,y)]. 21

For SSR, with the decreasing of scale parameter, image details become more prominent. Conversely, the dynamic range compression of gray value is more obvious. In order to overcome the shortcomings of SSR, Jobson et al. [22] present MSR which is an extension of SSR. The MSR output is a weighted sum of the outputs of several different SSR using different scale parameters (small, middle, and large). Mathematically, this is expressed by,

$$\begin{array}{l} r\left(x,y\right)=\sum_{i=1}^{k}\omega_{i}r_{i}\left(x,y\right)\;=\sum_{i=1}^{k}\omega_{i}[s\left(x,y\right)-l_{i}\left(x,y\right)]\;\\ \;=\sum_{i=1}^{k}\omega_{i}\{s\left(x,y\right)-\log[S\left(x,y\right)*G_{i}\left(x,y\right)]\}\\ \;\;\;\;\;\;\;\;\;=\sum_{i=1}^{k}\omega_{i}\{s\left(x,y\right)-\log[S\left(x,y\right)*{\text{Ke}}^{-\left(x^{2}+y^{2}\right)/c_{i}^{2}}]\} \end{array}$$ 22

where *k* designates the number of scales and usually takes the value of 3, {*ω*_*i*_}(i = 1,2,3) are the weights associated with the different scales and usually take the value of {1/3,1/3,1/3}, and the scale parameters {*c*_*i*_}(i = 1,2,3) usually take the value of {15,80,250}. When the image to be processed is RGB color image, three color bands are usually individually enhanced in MSR, which might create color distortion. To solve this problem, the Multi-Scale Retinex with Color Restoration (MSRCR) [36, 37] has emerged,

$$\begin{array}{l} r_{MSRCR}^{X}\left(x,y\right)=r^{X}\left(x,y\right)\cdot S_{X}^{'}\left(x,y\right)\\ =r^{X}\left(x,y\right)\cdot \log\{\text{C}S_{X}\left(x,y\right)/\left[S_{R}\left(x,y\right)+S_{G}\left(x,y\right)+S_{B}\left(x,y\right)\right]+1\} \end{array}$$ 23

where $S_{X}^{'}\left(x,y\right)$ denotes the color restoration factor of color band *X* (*X* = *R*,*G*,*B*), and C is a constant whose value is 125.

Due to the existence of logarithmic computation, image pixels tend to be negative in MSR and MSRCR and cannot provide a good visual effect after the directly inverse logarithmic computation. Therefore, it is necessary to translate and stretch the gray levels of the output images into the dynamic range of display device by the gain compensation technique [38]. This process can be represented as:

$$\begin{array}{l} r_{M}^{X}\left(x,y\right)=\alpha \cdot r_{MSRCR}^{X}\left(x,y\right)+\beta \\ \;=d_{\max}\cdot \left[r_{MSRCR}^{X}\left(x,y\right)/\left(r_{\max}-r_{\min}\right)-r_{\min}/\left(r_{\max}-r_{\min}\right)\right]\\ \;=d_{\max}\cdot \{[r_{MSRCR}^{X}\left(x,y\right)-r_{\min}]/\left(r_{\max}-r_{\min}\right)\} \end{array}$$ 24

where $r_{MSRCR}^{X}(x,y)$ is the color band *X* (*X* = *R*,*G*,*B*) of face image after MSRCR, $r_{M}^{X}(x,y)$ is the color band *X* (*X* = *R*,*G*,*B*) of face image after the gain compensation, α and β are the gain and the compensation respectively, *r*_max_ and *r*_min_ are maximum and minimum of the gray levels of the three color bands respectively, and *d*_max_ is the dynamic range of display device and usually takes the value of 255.

We mainly improve Retinex algorithm from two aspects. Firstly, we optimize the surround function. Secondly, we intercept the values in both ends of histogram of face image, determine the range of gray levels, and stretch the range of gray levels into the dynamic range of display device.

**Fig 5:**
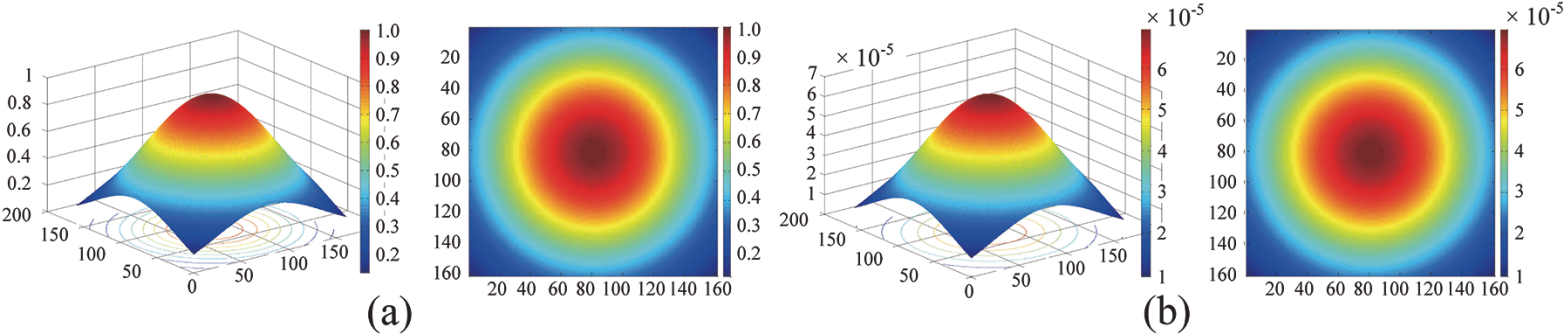
The three-dimensional and two-dimensional representations of Gaussian function used in MSRCR. (a) No normalization. (b) Normalization.

The traditional center/surround Retinex estimates the illuminant component by the convolution between the Gaussian function and the face image. We give the three-dimensional and two-dimensional representations of Gaussian function in Fig 5, where the points with the same value have the same Euclidean distance to the center. For illumination normalization of face image, the slowly changing illuminant components are often seen as the low frequency components. Furthermore, the structure of Gaussian function exhibit low pass filter characteristic. Therefore, the convolution between the Gaussian function and the face image can perform illuminant component extraction.

**Fig 6:**
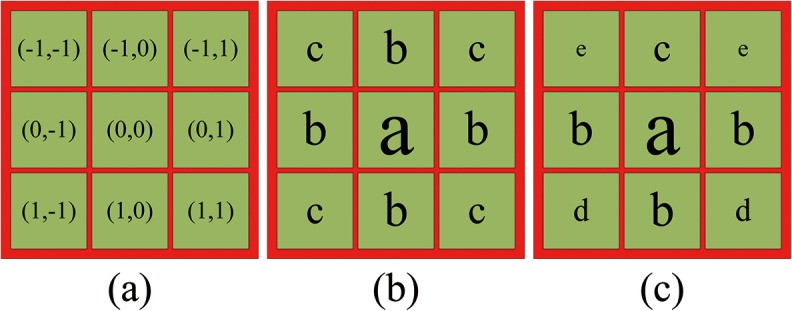
Central region’s coordinates of Gaussian and their corresponding values. (a) Coordinate. (b) The distribution of conventional Gaussian’s values. (c) The real situation.

The central region’s coordinates of conventional Gaussian and their corresponding values are shown in Fig 6A and 6B Gaussian convolution in the center/surround Retinex method is essentially a process of weighted average for the central pixel and its surrounding pixels. a, b, and c (a > b > c > 0) in Fig 6B are the weights whose values represent the confidence levels. Here, the confidence level means the pixel’s contribution to illuminant component estimation of the central pixel. The traditional Gaussian function makes center/surround Retinex method have an essential property: these pixels with the same Euclidean distance to the center have the same contribution to illuminant component estimation of the central pixel. Therefore, we can conclude that the traditional center/surround Retinex method holds the opinion that illuminant distribution is even within its specified image region. However, Gaussian convolution template with large size usually occupies most of the image, and the assumption of illuminant uniform distribution within this template is not realistic.

In reality, the pixels which are closer to the light source have the stronger illuminant components, and the ones which are away from the light source have the opposite characteristic. Therefore, we believe that Gaussian function for estimating the illuminant component should have directivity, thus the pixels whose illuminant components are stronger should have smaller weights and the ones with the opposite characteristic larger weights. It is assumed that the illuminant direction is from top to bottom. We adjust Gaussian function and get a new distribution about the central region’s values, as shown in Fig 6C where a, b, c, d, and e (a > b > c > d > e > 0) indicate the weights.

For improving the Retinex theory, we firstly introduce the parameters *t*_1_ and *t*_2_ (*t*_1_,*t*_2_ ≥ 1) to Gaussian function.

$$F(x,y)={\text{Ke}}^{-(t_{1}x^{2}+t_{2}y^{2})/c^{2}}.$$ 25

Eq. (25) generates three different cases according to the differences of *t*_1_ and *t*_2_.

Case 1: if *t*_1_ = *t*_2_ then

$$F(x,y)={\text{Ke}}^{-(t_{1}x^{2}+t_{2}y^{2})/c^{2}}={\text{Ke}}^{-(x^{2}+y^{2})/(c^{2}/t_{1})}.$$ 26

Case 2: if *t*_1_ ≠ *t*_2_ and 1 ≤ *t*_2_ < *t*_1_ then

$$F\left(x,y\right)={\text{Ke}}^{-\left(t_{1}x^{2}+t_{2}y^{2}\right)/c_{}^{2}}={\text{Ke}}^{-t_{2}\left(t_{1}x^{2}/t_{2}+y^{2}\right)/c_{}^{2}}\;={\text{Ke}}^{-\left(t_{1}x^{2}/t_{2}+y^{2}\right)/\left(c_{}^{2}/t_{2}\right)}$$ 27

Case 3: if *t*_1_ ≠ *t*_2_ and 1 ≤ *t*_1_ < *t*_2_ then

$$F\left(x,y\right)={\text{Ke}}^{-\left(t_{1}x^{2}+t_{2}y^{2}\right)/c_{}^{2}}={\text{Ke}}^{-t_{1}\left(x^{2}+t_{2}y^{2}/t_{1}\right)/c_{}^{2}}={\text{Ke}}^{-\left(x^{2}+t_{2}y^{2}/t_{1}\right)/\left(c_{}^{2}/t_{1}\right)}$$ 28

Comparing with the original Gaussian function, Eq. (26) has no essential differences in the distribution of values. Therefore, this case does not meet our requirement. Obviously, it can be transformed into each other between Eq. (27) and Eq. (28) through rotation and scale change. Therefore, the two equations can be simplified as:

$$F(x,y)={\text{Ke}}^{-(x^{2}+ty^{2})/c^{2}}(t>1).$$ 29

We further optimize Eq. (29) and obtain the modified Gaussian function:

$$F(x,y)=\{\begin{array}{cc} {\text{Ke}}^{-(x^{2}+ty^{2})/c^{2}} & \left(x\geq 0\right)\\ {\text{Ke}}^{-(x^{2}+ty^{2})/c^{2}} & \left(x<0\right) \end{array}.$$ 30

It should be noted that the Gaussian function in Eq. (30) is adapted to the illuminant direction at an angle of zero degrees (from top to bottom).

We have estimated the final illuminant direction *L*_*final*_, as is presented in Subsection 2.1. Let ${ℜ}_{\theta }(•)$ be the rotation function where *θ* is the angle of rotation. If *θ* is positive, the Gaussian function rotates *θ* degrees counterclockwise; if *θ* is negative, the Gaussian function rotates −*θ* degrees clockwise. The rotated Gaussian function can be defined as

$$F_{ℜ}(x,y)={ℜ}_{L_{final}}(F(x,y)).$$ 31

**Fig 7:**
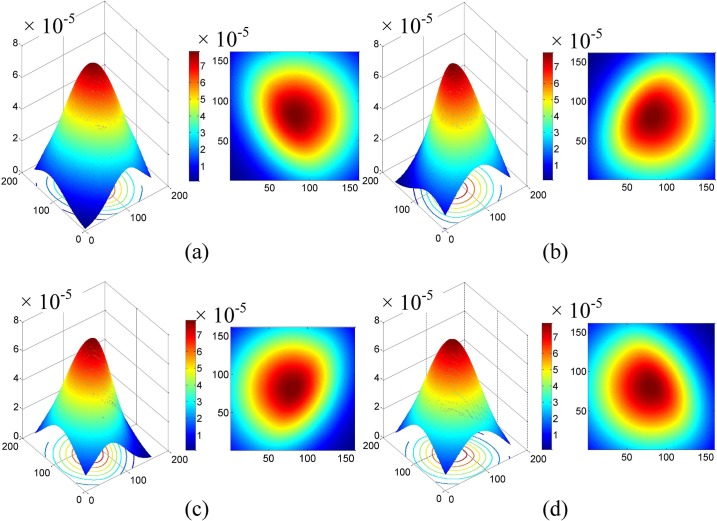
The three-dimensional and two-dimensional representations of Gaussian function used in our method. **(a)** 60°. **(b)** 120°. **(c)** −60°. **(d)** −120°.

For example, Fig 7 shows the three-dimensional and two-dimensional representations of rotated Gaussian function, where *L*_*final*_ is 60°, 120°, −60°, and −120°, respectively. As can be seen from Fig 7, according to the different illuminant distributions of face images, the improved Gaussian function with different directivities makes the pixels whose illuminant components are stronger obtain the smaller weights and the ones with the opposite characteristics have the larger weights.

Moreover, another shortcoming of traditional Retinex theory in Eq. (24) that *r*_max_ and *r*_min_ are just maximum and minimum of the gray levels in the image, respectively. After analyzing the MSR, Zhao [38] suggested that the selection of two intercept point in histogram is the key to the global luminance adjustment. The gain compensation technique used in Eq. (24) makes the values which have small probability and locate in both ends of histogram take up too much gray level. So these values which truly present the details of face images do not have enough gray level. From this we intercept the values in both ends of histogram, determine the range of gray levels, and stretch the range of gray levels into the dynamic range of display device. Interception rules can be expressed as:

$$r_{M}^{X}\left(x,y\right)=\{\begin{array}{c} r_{low}^{X}\;r_{M}^{X}\left(x,y\right)\leq r_{low}^{X}\;\\ r_{M}^{X}\left(x,y\right)\;r_{low}^{X}\;\leq r_{M}^{X}\left(x,y\right)\leq r_{up}^{X}\;\\ r_{up}^{X}\;r_{M}^{X}\left(x,y\right)\geq r_{up}^{X}\; \end{array}$$ 32

where $r_{up}^{X}$ and $r_{low}^{X}$ are the intercepted maximum and minimum in the histogram of the color band *X* (*X* = *R*,*G*,*B*) of face image, respectively. Let $r_{low}=\min\left\{r_{low}^{X}\right\}$ and $r_{up}=\max\left\{r_{up}^{X}\right\}$, the range of gray levels in intercepted image is [*r*_*low*_,*r*_*up*_]. Then, linear stretching can be implemented as following:

$$\begin{array}{l} r_{M-\text{final}}^{X}\left(x,y\right)=\alpha \cdot r_{M}^{X}\left(x,y\right)+\beta \\ \;=d_{\max}\cdot \left[r_{M}^{X}\left(x,y\right)/\left(r_{up}-r_{low}\right)-r_{low}/\left(r_{up}-r_{low}\right)\right]\\ \;=d_{\max}\cdot \{[r_{M}^{X}\left(x,y\right)-r_{low}]/\left(r_{up}-r_{low}\right)\} \end{array}$$ 33

**Fig 8:**
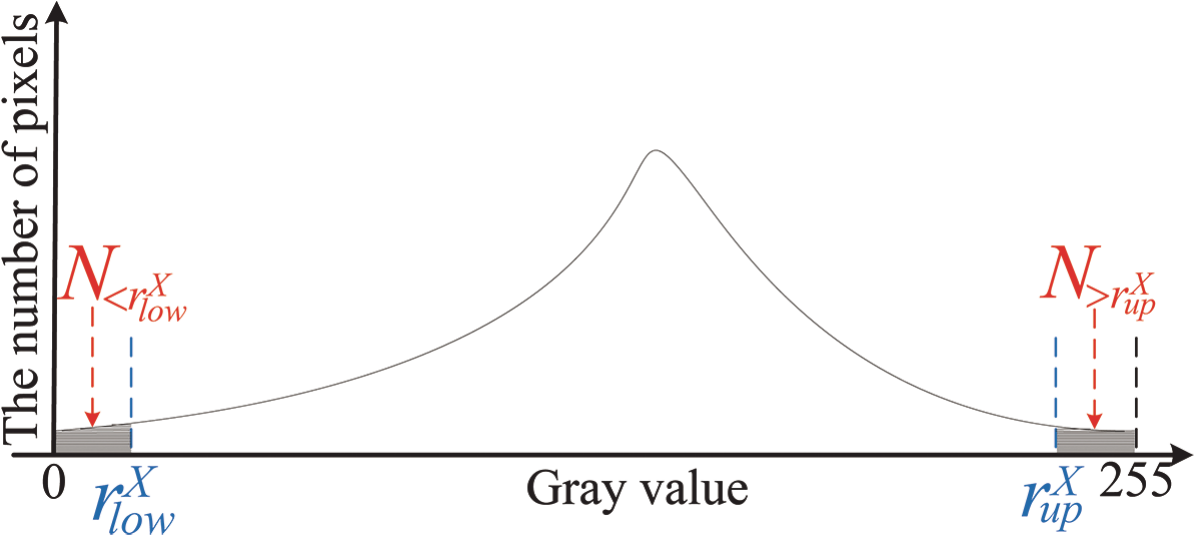
The schematic diagram for the rule of interception.

For determining $r_{up}^{X}$ and $r_{low}^{X}$, we adjust the method proposed in [38] and state the rule:

$$\min\;abs(N_{<r_{low}^{X}}+N_{>r_{up}^{X}}-N_{sum}\times 1\%)$$ 34

where $N_{<r_{low}^{X}}$ and $N_{>r_{up}^{X}}$ are the numbers of abandoned pixels locating in the left and right of the histogram, respectively, *N*_*sum*_ denotes the total number of pixels whose value is width × height. The detail about the interception can be seen in Fig 8.

## Experimental Results and Analysis

In this section, we test and verify our proposed method by several experiments on gray face images from the extended Yale face database B [39, 40] and color face images from CMU-PIE [41], since the two face databases are commonly used to evaluate the performance of illumination normalization. Under the same experimental condition, we compare the proposed method with Histogram Equalization (HE) [12], Local Normalization Technology (LNT) [14], Local Mean Map (LMM) of an image representing its low-frequency contents [15], Local Variance Map (LVM) carrying the image’s high-frequency components [15], LNT together with HE [15], Illumination Plane Subtraction (IPS) together with HE [16], Single-Scale Retinex (SSR) [21], Multi-Scale Retinex (MSR) [22], Multi-Scale Retinex with Color Restoration (MSRCR) [36, 37], and Local Binary Pattern (LBP) [24]. As our work focus on illumination normalization, only frontal face images without variations in head pose are considered and resized to 394×326 pixels. It should be noted that the parameters of Canny edge detector [T1 T2] and σ are set as [0.04 0.10] and 1.5, respectively. All the experiments are performed in Matlab2011 environment on a computer with an Intel (R) Core (TM)2 Duo CPU with a clock speed of 2.2 GHz, 2 GB RAM and Windows XP Professional.

Five experiments were carried out in our work. In the first part, we compare the different experimental results for different parameter *t* which is presented in Eq. (30). In the second part, the experimental results for the case of gray face images are described. Then, the third part describes the experimental results for the case of color face images. In the fourth part, for face images which are processed by different illumination normalization methods, we perform the feature point localization by Active Appearance Model (AAM) [42]. Finally, in the fifth part, we show the results of face recognition task, and present the performance measures used to evaluate the feasibility of our proposed method.

### 3.1 Choice of Parameter t

**Fig 9:**
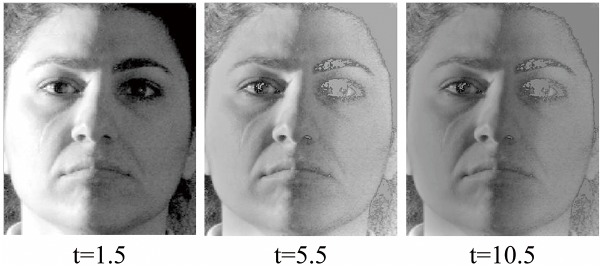
Comparison among three different sizes of parameter *t*. Reprinted from http://vision.ucsd.edu/~leekc/ExtYaleDatabase/ExtYaleB.html under a CC BY license, with permission from David Kriegman, original copyright 2001.

For the parameter *t* in Eq. (30), we aim to determine its best value in illumination normalization. We perform some comparisons among three different values of parameter *t* which are small, middle, and large, respectively, whereas the number of face images is 10. The experimental results of one sample are given in Fig 9 where the parameter *t* takes the value of 1.5, 5.5, and 10.5, respectively. Obviously, when the parameter *t* is 1.5, the effect of illumination normalization is satisfactory.

### 3.2 Experimental Results for Gray Face Images

**Fig 10:**
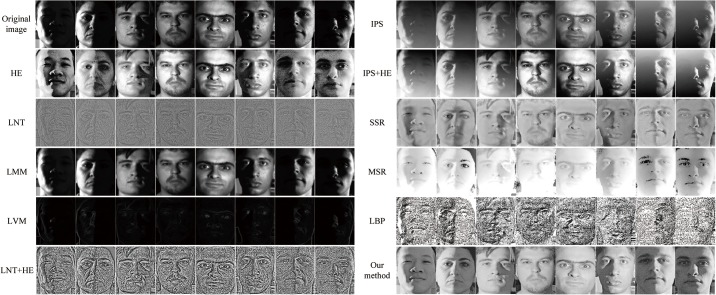
Comparison of different illuminant normalization methods for gray face images from the Yale face database B. Reprinted from http://vision.ucsd.edu/~leekc/ExtYaleDatabase/ExtYaleB.html under a CC BY license, with permission from David Kriegman, original copyright 2001.

To evaluate the effectiveness of the proposed method, gray face images from the extended Yale face database B are used in the second experiment. This database is set up for performance evaluation of face recognition and illuminant-processing algorithms under large variations in illumination and pose. In our work, 342 face images of 38 human subjects representing nine illuminant conditions (0° elevation) under frontal pose are employed. The human subjects comprise 10 individuals in the original Yale face database B and 28 individuals in the extended Yale face database B. It should be noted that the parameter *t* is 1.5 in this experiment. The left side of Fig 10 illustrates the original images in the databases on the first row, the images processed by HE on the second row, those processed by LNT on the third row, those processed by LMM on the fourth row, those processed by LVM on the fifth row, those processed by LNT + HE on the sixth row. The right side of Fig 10 illustrates those processed by IPS on the first row, those processed by IPS + HE on the second row, those processed by SSR on the third row, those processed by MSR on the fourth row, those processed by LBP on the fifth row, and those processed by our algorithm on the bottom row. Obviously, the results in Fig 10 prove that comparing with others, our proposed method can better recover uncertain contours of face images, more effectively remove the negative effect of illumination, more satisfactorily preserve the texture information of face images, and more successfully enhance the image contrast. Of course, all the advantages mentioned above are useful and important to the feature point localization and then for FR and FER.

### 3.3 Experimental Results for Color Face Images

**Fig 11:**
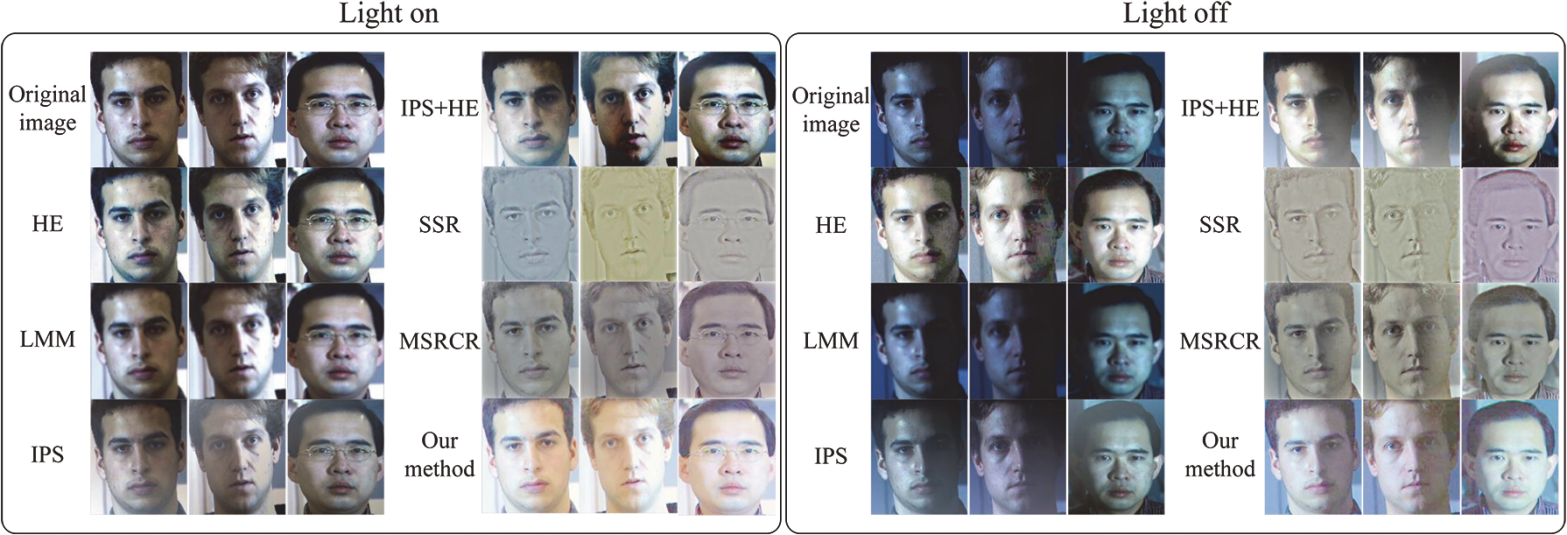
Comparison of different illuminant normalization methods for color face images from the CMU-PIE database. Reprinted from http://www.ri.cmu.edu/research_project_detail.html?project_id=418&menu_id=261 under a CC BY license, with permission from Takeo Kanade, original copyright 2003.

Between October 2000 and December 2000, Sim et al. [41] collected a total of 41,368 facial images of 68 people and built the CMU-PIE database. All the images were captured in the CMU 3D Room, and each subject was imaged across 13 different poses, under 43 different illumination conditions, and with 4 different expressions. For our third experiment, we employ the frontal facial images (c27 in the numbering scheme) with significant illumination variation and the room lights switched on and off. It should be noted that all the experimental images are colorful and the parameter *t* is also 1.5 in this experiment. Otherwise, for the facial images of three color bands (R, G, and B), we firstly apply the illuminant normalization methods to one color band and then combine the three results into the final color image. Fig 11 shows some representative results processed by some different illuminant normalization methods and ours. From this figure, it can be seen that the visual performance of our method is superior to that of the other methods.

### 3.4 Feature Point Localization

**Fig 12:**
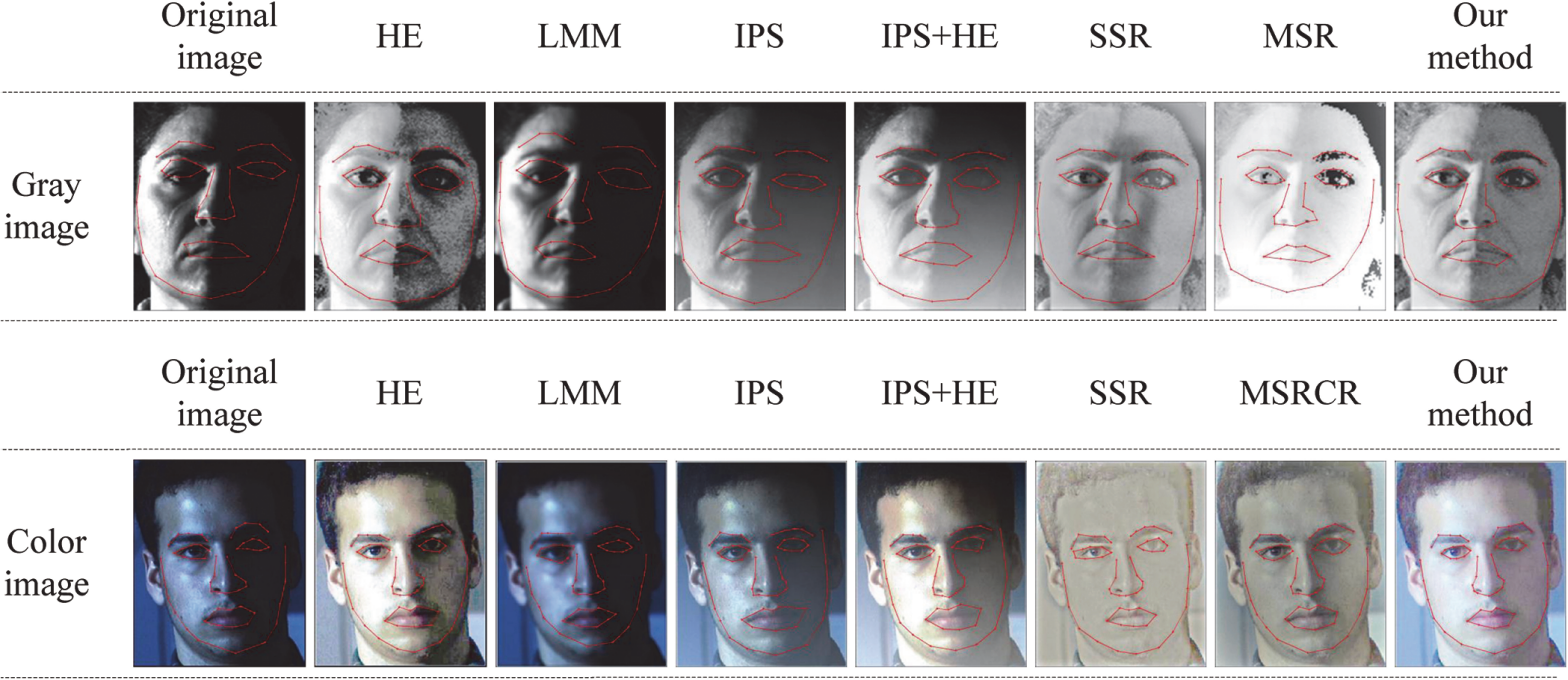
The analysis of feature point location. Reprinted from http://vision.ucsd.edu/~leekc/ExtYaleDatabase/ExtYaleB.html under a CC BY license, with permission from David Kriegman, original copyright 2001.

It is known that texture information and structural characteristic are two important factors for FR or FER. The results of the above two experiments have shown our method can effectively remove the negative effect of illumination and satisfactorily preserve the texture information of face images. In this experiment, we aim to test and verify if method is helpful to supply the structural characteristics to the subsequent studies. Facial feature point located by AAM is a popular and important factor to represent the structural characteristic of face image. Therefore, for face images processed by different illuminant normalization methods, we compare the effects of feature point location in this experiment. We select the face images with uniform illumination to construct the training set of AAM. Furthermore, it should be noted that the training set is rebuilt for the different samples in our experiments. That is to say, for one location experiment, the training set and testing set are from the same person. Some samples’ effects of feature point location are shown in Fig 12.

The results in Fig 12 demonstrate the advantage of our proposed method. In order to further analyze the effect of feature point location, we assume that the coordinate of feature point A marked by human is (*x*_*h*_,*y*_*h*_) and the coordinate of feature point A marked by computer is (*x*_*c*_,*y*_*c*_). The error *r*_*A*_ is defined as the Euclidean distance between the two coordinates.

$$r_{A}=\sqrt{{\left(x_{c}-x_{h}\right)}^{2}+{\left(y_{c}-y_{h}\right)}^{2}}.$$ 35

**Fig 13:**
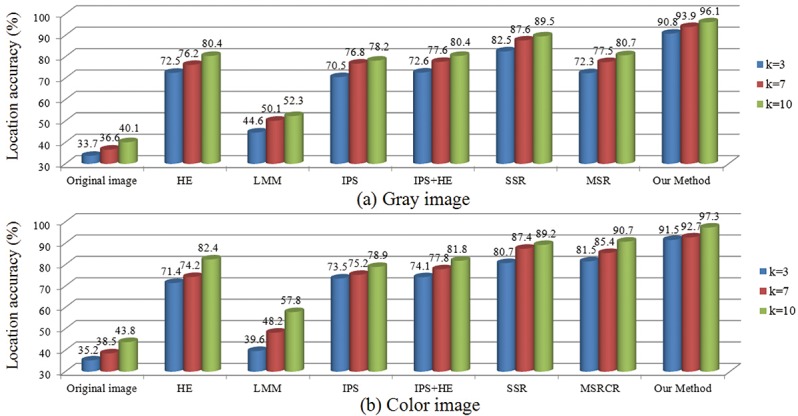
The location accuracies for different methods. (a) Gray image. (b) Color image.

Let *k* be the error that can be allowed. When *r*_*A*_ is less than or equal to *k*, we assume that the location is successful, otherwise it is a fail. A total of 2320 feature points of 40 face images (20 gray images and 20 color images) were selected for the experiment. It should be noted that the participants have different age, skin color, and gender. When *k* is 3, 7, and 10, we calculate the location accuracies of different methods (see Fig 13). As shown in Fig 13, as *k* increases, the location accuracies of all the methods also increase. Meanwhile, the location accuracy of our method always has the highest value.

### 3.5 Face Recognition

In order to further verify the superiority of our proposed method, we perform face recognition in the two databases based on feature point localization. For each sample of the extended Yale face database B, three face images with uniform illumination are selected to the training set, and the rest with uneven illumination constitutes the testing set. So the training set consists of 114 face images. For each sample of CMU-PIE database, we employ four frontal face images (c27 in the numbering scheme) with uniform illumination to constitute the training set, and the rest with uneven illumination constitutes the testing set. So the training set consists of 272 face images. Note that all the selected face images of the two databases are in a neutral emotional state.

In a face image, the distance between two points can be expressed as:

$$d_{ij}=\sqrt{{\left(x_{i}-x_{j}\right)}^{2}+{\left(y_{i}-y_{j}\right)}^{2}}\;\left(0<i<j\leq 58\right).$$ 36

All the distances constitute one set {*d*_*ij*_} (0 < *i* < *j* ≤ 58). Let *d*′ be the maximum value of the set, that is

$$d^{'}=\max\left\{d_{ij}\right\}\;\left(0<i<j\leq 58\right).$$ 37

We normalize every *d*_*ij*_ and get set

$$D=\left\{d_{ij}/d^{'}\right\}\;\left(0<i<j\leq 58\right).$$ 38

**Fig 14:**
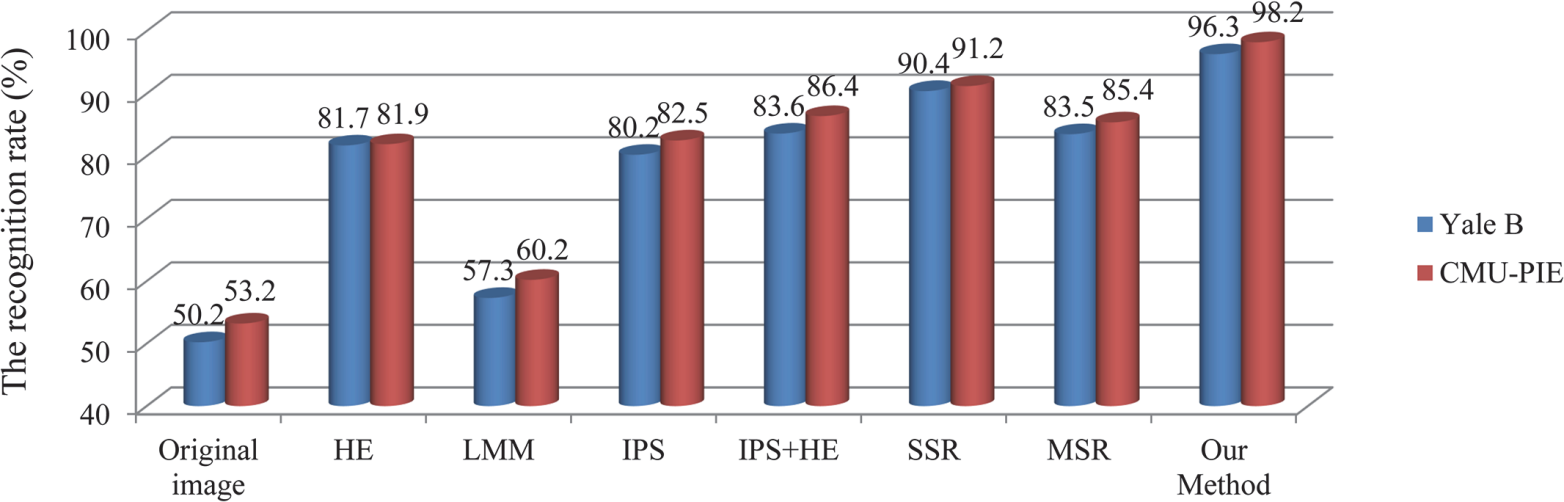
Performance comparisons of different methods.

Note that *D* is the input feature of our face recognition task. After calculating the input features to all face images, we reference [30] and apply Nearest Neighbor Decision (NND) to recognize every test sample’s identity. We compare our method with other well-known methods to prove its improvement. As shown in Fig 14, our proposed method can reach the highest recognition rate compared to the others for both the extended Yale face database B and CMU-PIE database. The satisfactory recognition rate obtained by our proposed method further shows that our algorithm is capable to remove the negative effect of illumination and preserve the texture and structural information of face image.

## Conclusion

In this paper, a new method for illumination normalization of face image has been proposed. In our method, we firstly estimate the given face image’s illuminant direction based on local region complexity analysis and average gray value. Then, with the help of the calculated illuminant direction, we improve the original Retinex algorithm by redefining the surround function and adjusting global luminance. Our original method creates promising perspectives in illumination normalization for face images. The experimental results for gray and color face images show the significant advantages of the proposed method over the existing ones.

**Fig 15:**
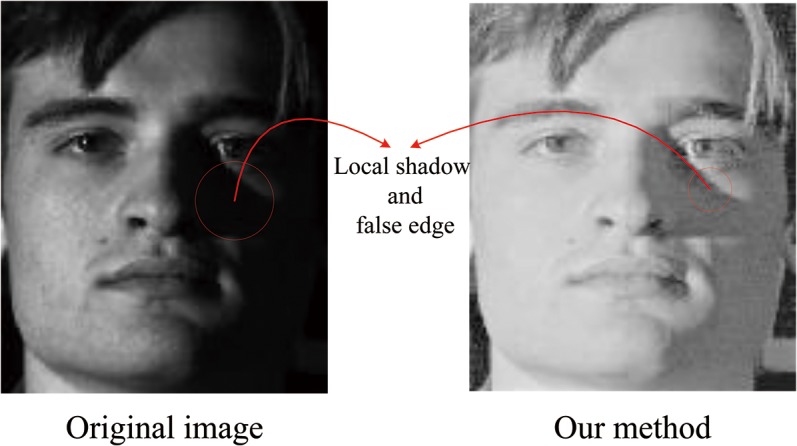
Local shadow and false edge around the nose. Reprinted from http://vision.ucsd.edu/~leekc/ExtYaleDatabase/ExtYaleB.html under a CC BY license, with permission from David Kriegman, original copyright 2001.

Our proposed method aims to improve the facial image illumination condition and could be used in the subsequent FR and FER processes. Although the proposed method performs well on overall illumination normalization, it fails to completely eliminate the local shadows and false edges around the nose, which is the most challenging task for all of the illuminant normalization methods. Fig 15 illustrates this problem. Therefore, the authors will devote research effort to overcome this limitation in the future.

The authors would like to thank the providers of the extended Yale face database B and the CMU-PIE.
